# Treatment of Stricture of the Female Uretha

**Published:** 1893-11

**Authors:** 


TREATMENT OF STRICTURE OF THE FEMALE URETHRA.—Dr. W. A. Meisels thinks that more attention should be paid to stricture of the urethra in females, as he is certain that numerous cases of vesical neurosis and dysuria and the associated reflex neuralgias, which at present are treated symptomatically, can be cured by urethral treatment. As regards the operative method of treating urethral strictures in females, he believes that gradual dilatation with sounds (preferably Hegar's sounds), in several sittings, usually brings about the desired result, and that it is rarely necessary to resort to the knife. To prevent recurrences it is necessary to introduce sounds from time to time. The use of the endoscope for diagnosing the nature of extent of the urethral lesion is of great advantage.—*Western Medical Reporter.*

